# PassTCN-PPLL: A Password Guessing Model Based on Probability Label Learning and Temporal Convolutional Neural Network

**DOI:** 10.3390/s22176484

**Published:** 2022-08-29

**Authors:** Junbin Ye, Min Jin, Guoliang Gong, Rongxuan Shen, Huaxiang Lu

**Affiliations:** 1 Institute of Semiconductors, Chinese Academy of Sciences, Beijing 100083, China; 2 University of Chinese Academy of Sciences, Beijing 100049, China; 3 Semiconductor Neural Network Intelligent Perception and Computing Technology Beijing Key Laboratory, Beijing 100083, China; 4 Materials and Optoelectronics Research Center, University of Chinese Academy of Sciences, Beijing 100083, China; 5 Collage of Microelectronics, University of Chinese Academy of Sciences, Beijing 100089, China

**Keywords:** password guessing, temporal convolutional neural network, password label

## Abstract

The frequent incidents of password leakage have increased people’s attention and research on password security. Password guessing is an essential part of password cracking and password security research. The progression of deep learning technology provides a promising way to improve the efficiency of password guessing. However, the mainstream models proposed for password guessing, such as RNN (or other variants, such as LSTM, GRU), GAN and VAE still face some problems, such as the low efficiency and high repetition rate of the generated passwords. In this paper, we propose a password-guessing model based on the temporal convolutional neural network (PassTCN). To further improve the performance of the generated passwords, we propose a novel password probability label-learning method, which reconstructs labels based on the password probability distribution of the training set and deduplicates the training set when training. Experiments on the RockYou dataset showed that, when generating $10^{8}$ passwords, the coverage rate of PassTCN with password probability label learning (PassTCN-PPLL) reached 12.6%, which is 87.2%, 72.6% and 42.9% higher than PassGAN (a password-guessing model based on GAN), VAEPass (a password-guessing model based on VAE) and FLA (a password-guessing model based on LSTM), respectively. The repetition rate of our model is 25.9%, which is 45.1%, 31.7% and 17.4% lower than that of PassGAN, VAEPass and FLA, respectively. The results confirm that our approach not only improves the coverage rate but also reduces the repetition rate.

## 1. Introduction

With the development of the Internet, people’s lives are becomign increasingly integrated with various digital services. The identity authentication technology ensures that people have highly valued information security. It is also a research hotspot in the field of security. Although new authentication technologies have been proposed in recent years [1,2,3], password is still the most widely used authentication method due to its simplicity, flexibility, and ease of use. It is foreseeable that passwords will be an essential authentication method for a long period of time [4]. However, the use and storage of passwords is not always secure. The frequent incidents of password leakage have increased people’s attention and research on password security. The human brain has limited memory, and it tends to use simple and memorable forms for password setting, which leads to the existence of many weak passwords [5]. As people use more and more passwords, similar passwords are inevitably used in different services [6,7]. However, the ever-increasing password attack technology and computing power exacerbate the security threats faced by passwords. Therefore, it is of great practical significance to study password strategy and password security.

There are two common ways of evaluating password security. One is a password complexity assessment [6,8]; the other is the cracking difficulty assessment [9]. The basis of password complexity assessment is the user password creation rules, such as whether the password is long enough, whether it contains multiple characters, and whether there is a palindrome or repetition. At present, most websites use this method to provide strength assessment prompts to users when setting passwords, but this artificially set rule is relatively simple and solid. Although some passwords match the conditions for strong passwords, there may be special rules that adversaries can easily crack during password attacks. Therefore, an assessment of the difficulty of cracking can more truly reflect the strength of a password. Password generation is a core part of password cracking. Users’ habits and hidden patterns should be studied and excavated when setting passwords to obtain a more accurate password generation model and lay the foundation for subsequent research on password cracking and password security assessments [8].

Early password generation creates passwords through a dictionary and variant rule enumeration, such as the famous password cracking tools HashCat [10] and John the Ripper [11]. These methods require the manual formulation of transformation rules, so the generation effect is limited by personal experience. Later, scholars introduced the Markov model [12] and probabilistic context-free grammar (Probabilistic Context-Free Grammar, PCFG) [13] to password generation; the Markov model learns the dependence of characters to establish a password’s probability model; PCFG learns the structural distribution of passwords from the training set, and depicts this distribution law in the form of probability. These two methods are theoretical and systematic, so the effect is better than generating passwords through variant rule enumeration. Subsequently, scholars have also proposed improved methods for the password generation model based on Markov and PCFG [14,15,16,17]. The more representative one is the Ordered Markov Enumerator (OMEN) algorithm proposed by Dutirmuth et al. [14] in 2015. This can enumerate and generate passwords in descending order of probability, which significantly improves the efficiency of subsequent password cracking. Zhang Mengli et al. [15] combined PCFG and Markov to form a hybrid attack model SPSR. When examining the attack effect under the same number of guesses, the SPSR model can crack 40% to 50% more passwords than the Markov-based model and about 20% more passwords than the PCFG-based model.

The development of deep learning in recent years has provided a new approach to password generation. In 2016, Melicher et al. [18] proposed a password generation model based on Recurrent Neural Network (RNN) and obtained a higher cracking rate than PCFG and Markov models. In 2018, Hitaj et al. [19] first proposed PassGAN, a password-generation model based on Generative Adversarial Network (GAN). A recent article [20] shows that the variational auto-encoder (VAE) also performs well in password-guessing. Zhou et al. [21] integrated the user’s personal information into the Long-Short Term Memory (LSTM) network for directional password-guessing, and the success rate was significantly higher than that of Markov and PCFG in the same scenario. Scholars also combine neural network models with traditional models to further improve the efficiency of password-guessing. The PL model proposed by Liu et al. [22] combines PCFG and LSTM, takes the basic structure of the password as the network input, and uses LSTM to predict the following basic structure. This model improves the password training from character-based to word-based, thereby significantly improving the hit rate of a single dataset. Wang Ding et al. [23] combined RNN with PCFG and proposed models such as PR and PR+, whose cracking rate was significantly higher than that of a single model.

The neural network models used for password generation at present are mainly RNN (or other RNN variants, such as LSTM, GRU), GAN and VAE. Although these password models have a higher performance than the traditional ones, there are still some problems, such as the low efficiency and high repetition rate of the generated password. RNNs only calculate the next step after the end of the previous time-step, which is unsuitable for large-scale parallelism. Therefore, the password-generation efficiency of RNN is relatively low. The high repetition rate of the generated passwords is also a common problem in neural network models. For example, as the number of passwords generated by PassGAN [19] reaches $10^{8}$, only 53% of the passwords are unique, and the repetition rate increases as the sizes grow, which is a prominent disadvantage for subsequent password-cracking. Wang Ding et al. [23] proposed a model that can generate all unique passwords with a higher probability than a certain probability by maintaining the password prefix and the password prefix probability table during password generation. However, all possible characters must be renumerated for each password prefix, which is very costly in terms of time and space, to generate a new password prefix or a new password.

As neural network models still have the problems of a low coverage rate and high repetition rate in the field of password-guessing, our work aims to solve current neural network models’ problems for password generation. The major contributions of our work are as follows:

1. We propose a password-guessing model based on a temporal convolutional neural network called PassTCN, which obtains a better performance than the password-guessing model based on LSTM, GAN and VAE;
2. We propose a novel training method for password-guessing, which can further improve the performance of the generated passwords. It reconstructs labels based on the password probability distribution of the training set and deduplicates the training set when training.

The rest of this paper is organized as follows. Section 2 provides a brief overview of the related work on temporal convolutional neural networks. Section 3.1 illustrates the architecture of our password-guessing model, PassTCN. Section 3.2 proposes our probability lable learning method, which increases the coverage rate and reduces the repetition rate of password generation. We validate the PassTCN-PPLL and discuss the hyperparameter used for the probability lable learning method in Section 4. Finally, we summarize our work in Section 5.

## 2. Related Work

Recurrent neural networks are used to deal with sequence issues because the self-regression structure of RNNs can represent timing series well. RNNs have achieved incredible success in a variety of problems. However, they are not suitable for large-scale parallelism. Therefore, scholars have introduced Convolutional Neural Networks (CNNs) to sequence problems. The traditional convolutional neural network was generally not considered to be appropriate for the modeling of timing problems, mainly due to the limitations in the size of the convolution kernel, which cannot capture the long-term dependent information. In 2018, Shaojie Bai et al. [24] described a generic temporal convolutional network (TCN) architecture and found that TCN achieved higher accuracy in 9 of 11 typical timing tasks compared to RNN and LSTM.

**Figure 1:**
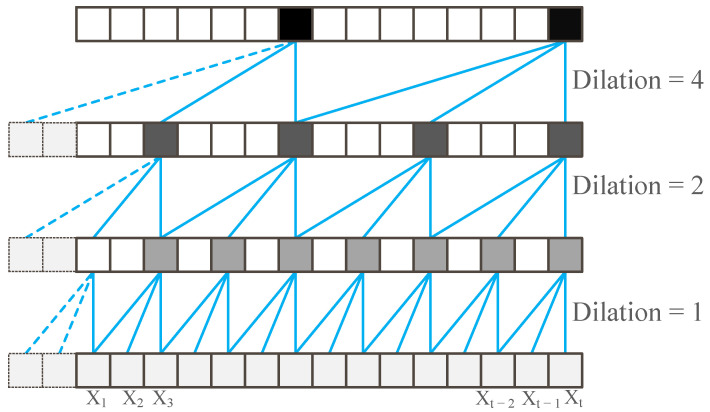
The dilated causal convolution.

TCN combines causal convolutions and dilated convolutions, as shown in Figure 1. Assuming the filter $F=(f_{1},f_{2},\ldots ,f_{K})$ and the sequence $X=(x_{1},x_{2},\ldots ,x_{t})$, then the causal convolution at the $x_{t}$ is:

(1) $$\left(F*X\right)\left(x_{t}\right)=\sum_{i=1}^{K}f_{i}x_{t-K+i},$$

The causal convolution does not consider future information, and the value at time *t* of the current layer only depends on the value at time *t* of the next layer and the value before this. Suppose the dilation factor at $x_{t}$ is *d*; then, the dilated convolution is:

(2) $$\left(F{*}_{d}X\right)\left(x_{t}\right)=\sum_{i=1}^{K}f_{i}x_{t-(K-i)d},$$

TCN changes the receptive field to obtain long effective history information with an adjustable length by increasing the number of layers and changing the dilation factor *d*. When the dilation factor *d* is 1, it degenerates into ordinary convolution. In practice, the dilation factor exponentially increases as the size of network layers increases. To allows for the network see further history, we have to build a deeper network or larger filters, which leads to the problem of gradient vanishing and is hard to train. TCNs introduced the residual block to solve these problems. The residual block adds the output of the residual function to the input. Therefore, we need a one-dimensional, fully convolutional network (FCN), which assures that the subsequent layers are the same size as the previous ones.

## 3. Method and Model Structure

### 3.1. Password Guessing Model Based on TCN

**Figure 2:**
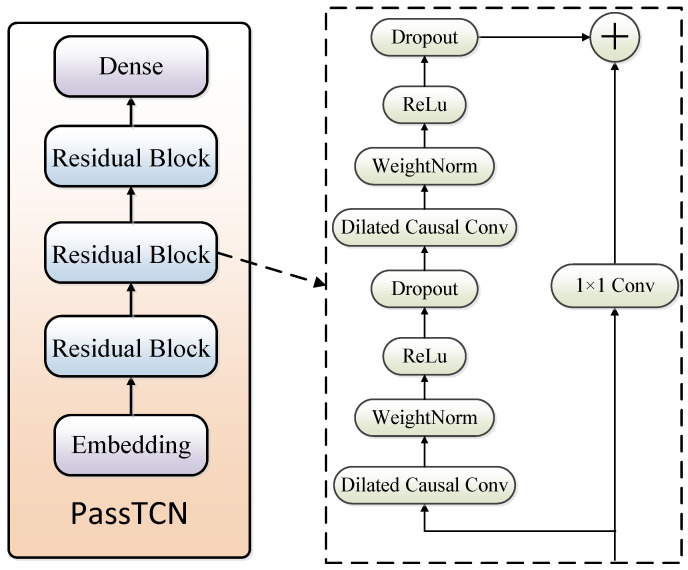
Password-guessing model based on TCN.

We propose a password guessing model based on a temporal convolutional neural network, called PassTCN, as shown in Figure 2. The network consists of an encoding module, a dense module and three groups of residual modules. The embedding block and the decoding block are two fully connected layers with shared weights. Firstly, the original ASCII-coded password is encoded into a 96-dimensional column vector by One-Hot (95 printable ASCII characters plus a special character ‘∖n’ with ASCII code 10 indicates the end of the password). Since One-Hot Encoding cannot effectively represent the association between characters (such as the association between upper-case and lower-case characters), we extend the 96-dimensional column vector to 256 dimensions through the embedding layer to better express the correlation between passwords’ characters. The output of the residual block is restored to One-Hot encoding by the decoding block. The residual block can more fluently create the forward and backward propagation of network information and retain the input features to a certain extent. All dilated causal convolutions in the residual block are a one-dimensional convolution kernel with a non-zero dilation factor. The dilation factors in these three residual blocks are 1, 2, and 4. With the increase in the dilation factor, the network can see further and learn the internal association between longer-length passwords. The dilated causal convolution layer is followed by the weight normalization layer, whose function is to decouple the weight vector into parameter vector *V* and scalar *g* on the Euclidean norm and its direction, and then optimize these two parameters. Weight normalization can provide the network with better convergence and stronger robustness. The dropout layer is a trick for neural network training. It randomly ignores nodes in each training batch with a certain probability, i.e., the activation value of a neuron stops working with a certain probability *P* during forward propagation. Dropout layers also dramatically enhance the network’s robustness and convergence.

### 3.2. Password Probability Label Learning Method

Our expectation to the password-guessing model is that the model can learn the features of passwords in the training set, grasp the habits of people setting passwords, and generate more new passwords in the testing set but not in the training set. We also aim to generate passwords that are as unique as possible to ensure a more efficient password cracking and password security evaluation. However, the public password dataset shows that there is lots of repetition between different users when setting passwords. The model will generate data that approximate the original sample distribution by learning the distribution of the dataset. Therefore, high-probability passwords are more frequently generated when generating passwords. The new passwords generated by the model must contain many duplicate passwords if we train the network directly using the origin dataset, as described in Section 4.2. A common approach is to deduplicate the password dataset, which inevitably loses some information. Therefore, we propose a learning method that can reduce the repetition rate without losing the probability distribution information in the data set.

**Figure 3:**
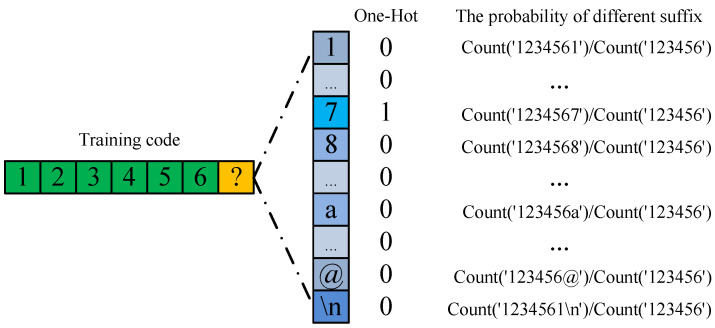
One-Hot Encoding and the probability of different password suffixes.

Before describing this method in detail, let us focus on the training process. We need to encode the password before it is sent to the network so that the computer can recognize it. The password is usually encoded with One-Hot Encoding, as shown in Figure 3. Assume that when the network trains the last digit of the password ‘1234567’, ‘7’ is the ground-truth label. If the password adopts One-Hot Encoding, only the position of label ‘7’ is 1, and all other characters are 0. According to the formula of Cross-Entropy Loss Function:

(3) $$loss=-\sum_{i}^{k}y_{i}log\left(p_{i}\right),$$

Therefore, only the ground-truth label ‘7’ is involved in the calculation, which ignores the relationship between the ground-truth label and other characters. This leads to useful knowledge not being learned, meaning that the model will be more arbitrary, and the generalization performance will be poor. In practice, we can calculate the probability of different characters under the known prefix by counting the passwords in the training set. Count(prefix) represents all passwords with the prefix string.

Szegedy et al. [25] first proposed a label-smoothing method to improve the drawbacks of One-Hot Encoding. The idea of label smoothing is to replace the original One-Hot hard label that is not 1 or 0 with a soft label and add random noises to each dimension to tell the model that the sample label has a certain probability of being incorrect. Label smoothing can alleviate the arbitrary problem of the model and has an anti-noise ability. However, adding random noise cannot reflect the relationship between labels, so it makes limited improvements to the model and even risks overfitting.

Based on the probability distribution information of passwords, we propose a learning method for password labels and construct unique password labels according to the probability distribution of passwords in the training set. First, we count the probabilities of different characters under the known password prefixes in the training set and then construct password labels according to the probabilities. Assuming that prefix is any possible password prefix, the ground-truth label of the next character is $y_{i}$. We can obtain the probability of the next arbitrary character *c* as follows:

(4) $$P\left(c|prefix\right)=\frac{Count(prefix+c)}{Count(prefix)},$$

Then, the password’s probability label *L* is:

(5) $$L=\left\{L\left(c|prefix\right)\right\}=\left\{P\left(c|prefix\right)\times \left(1-\alpha \right)+\sigma \left(c=y_{i}\right)\times \alpha \right\},$$

where α is a hyperparameter, a balance coefficient of the probability distribution of the password in the training set and the One-Hot Encoding value, which takes values [0, 1]. σ is Dirac delta, which equals 1 for $c=y_{i}$ and 0 otherwise. We also assign a minimal probability value to characters that never appear under the current password prefix. After constructing the password label *L*, we deduplicate the training set. If α=1, $L=\{\sigma \left(c=y_{i}\right)\}$ is the same as One-Hot Encoding. At this time, all passwords have the same probability, equivalent to simply deduplicating the training set. If α=0, L={P(c|prefix)} is the probability distribution of the password in the training set. When the α value is [0, 1], the label at this time is equivalent to a reduction in the password probability distribution in the training set while increasing the proportion of the ground-true label by α. The password label created by this method has the advantage of smoothing the label and represents the relationship between the ground-truth label and other characters, without losing information after deduplicating the training set. Our method can significantly improve the model’s performance, as shown in Section 4.3.

**Figure 4:**
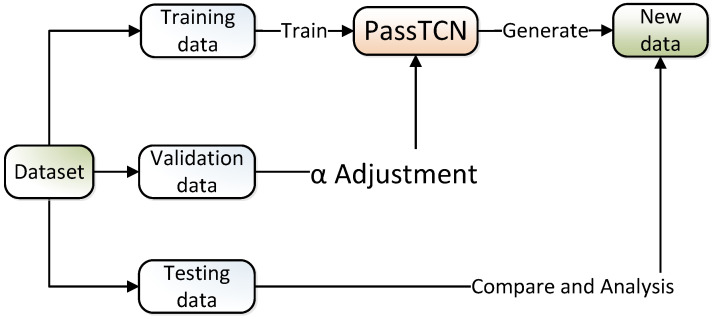
Verification process of hyperparameter α.

To better determine the value of the hyperparameter α, this paper uses the five-fold cross-validation method. The process is shown in Figure 4. We randomly divided the RockYou dataset into 8:2 and then randomly split the larger one into five parts. Choose one of the five pieces to be the validation dataset; the remaining four copies are the training set. In this way, we can obtain five groups of experiments with different datasets and calculate the arithmetic mean as the final result.

## 4. Experiment and Discussion

### 4.1. Dataset and Evaluation Metric

The dataset used in this paper is the password dataset leaked by RockYou, a famous social networking website, in 2009. There are 32,603,388 plaintext passwords in this password dataset. Considering that the characters used in passwords are generally printable ASCII characters, we cleaned up 18,038 unqualified passwords. Additionally, the standard password length requirement of the website is [6, 32], so we can clean 1,407,508 other passwords beyond this range. Finally, the dataset resulted in a total of 31,177,842 passwords, and the number of unique passwords is 14,048,038.

We hope the password generated by the model is as unique as possible and new passwords can be created that are in the testing set and not in the training set. Therefore, there are two main evaluation metrics for generating passwords:

(6) $$Repetition\;Rate=(1-\frac{New_{unique}}{New})\times 100\%,$$

(7) $$Coverage\;Rate=(1-\frac{New_{unique}\in \left(Test-Train\right)}{Test-Train})\times 100\%,$$

where New and $New_{unique}$ represent the number and the unique number of generated passwords. Test−Train is the unique number of passwords in the testing set and not in the training set.

We randomly divide the RockYou dataset into a training set (24,942,273 passwords, 11,655,200 unique passwords) and a testing set (6,235,569 passwords, 3,537,572 unique passwords) according to a ratio of 8:2. We train the model in the training set and compare the result with that of the testing set to calculate the generated password’s repetition rate and coverage rate.

We compare models in this paper with four classic password guessing models, OMEN [14], FLA [18] (which is based on LSTM), VAEPass [20] and PassGAN [19]. The configuration and dataset of these models are shown as follows:

- **OMEN**: We used the code in [26] to run a 4-ngram OMEN with the training set we used.
- **FLA**: Since the dataset used in [18] is a relatively small, specific testing dataset, we trained a three-layer FLA under the dataset we used, to allow for a better comparison with other models. We did not convert the training set (such as removing symbols or transforming all characters to lowercase) because this may lose some information. The hidden size of LSTM is 256, and we used the dropout layer between each LSTM layer. We trained the FLA for 20 epochs at batch size 512.
- **PassGAN**: The dataset segmentation ratio of RockYou used by PassGAN [19] was the same as 8:2 in this paper, and there was little difference in the size of the dataset after segmentation. The main difference is that PassGAN only retains passwords with a length of [5, 10]. Considering the general password-setting requirements in [6, 32] and the difficulty of cracking short passwords by brute force, the password length chosen in this paper was more reasonable. Therefore, we used the result in [19].
- **VAEPass**: We instantiated the model in [20] where the core module for constructing the VAE encoder and decoder was the Gated Convolutional Networks [27] (GCNN). The number of encoders and decoders was 2 and 1, respectively. Other model parameters were an input sequence length of 12, hidden layer of 256, latent space of 64, and convolution kernels of 5. We trained VAEPass for 20 epochs with a batch size of 128.

To clarify the influence that the hyperparameter α has on the password-probability label-learning method of password generation, we designed the cross-validation experiment in Section 3.2. Cross-validation is a very general approach that can be applied to almost any statistical learning method [28]. It divides the data into *k* folds of approximately equal size, of which k−1 folds are used for training, and the remaining fold is used for validation. This procedure was repeated *k* times, and a different fold was treated as a validation set each time. Then, we trained the model on these different training sets with the same parameters, except the hyperparameter α. This process results in *k* estimates of the validation score, $S_{1},S_{2},\ldots ,S_{k}$. The *k*-fold score of the hyperparameter α was calculated by averaging these values,

(8) $$Score_{\left(\alpha \right)}=\frac{1}{k}\sum_{i=1}^{k}S_{i}.$$

Finally, the optimal value of the hyperparameter α can be verified by comparing the model performance scores. Considering the hardware resources and model training time, we set the *k* value to 5 to confirm the optimal value of α. This method could reduce the differences caused by randomly divided datasets. Each piece of data from the 5-fold cross-validation dataset is detailed, as shown in Table 1:

All experiments were performed on a workstation with a Titan XP GPU. Table 2 lists the detailed configuration used in the experiment:

### 4.2. Password Guessing Model Based on TCN

TCNs can take inputs of arbitrary input lengths, just like RNNs. However, a practical implementation generally takes a fixed length of the sequence and divides the whole input sequence into historical and effective lengths. The effective length is involved in calculating the loss function in training. In contrast, the historical length is not involved in calculating the loss function, but provides additional historical information to the model to improve the final effect. Since the password length is in [6, 32], and 99.9% is in [6, 20], we set the total input length to 40 and the effective length to 20. The network architecture and number of parameters in each layer are shown in Table 3:

We trained the model for 20 epochs in the RockYou dataset, generated $10^{8}$ passwords and compared the results with other models. The experimental results are shown in Table 4.

As illustrated in the experimental results above, the coverage rate of PassTCN is 12.28%, which is the highest among all models, while that of OMEN is 9.89%, that of VAEPass is 7.30%, that of PassGAN is 6.73% and that of FLA is 8.82%. OMEN generates passwords with a repetition rate of 0 because OMEN enumerates passwords in the order of probability. However, all other network models have high repetition rates, especially PassTCN and PassGAN, which reach about 50%. At every training epoch, we set the weight of the hidden layer of LSTM modules to zero. However, this weight will deviate from the zero point as the sampling progresses, which may be why the repetition rate of FLA is relatively lower. Generative models aim to generate a similar distribution to the training set. There are many repeated passwords in the password training set, so these network models inevitably have a high repetition rate. Furthermore, the repetition rate becomes even higher when the sample size is larger. To improve the performance of the generated passwords, we propose a password-probability label-learning method to reduce the repetition rate.

### 4.3. Hyperparameter α for Password-Probability Label-Learning Method

**Figure 5:**
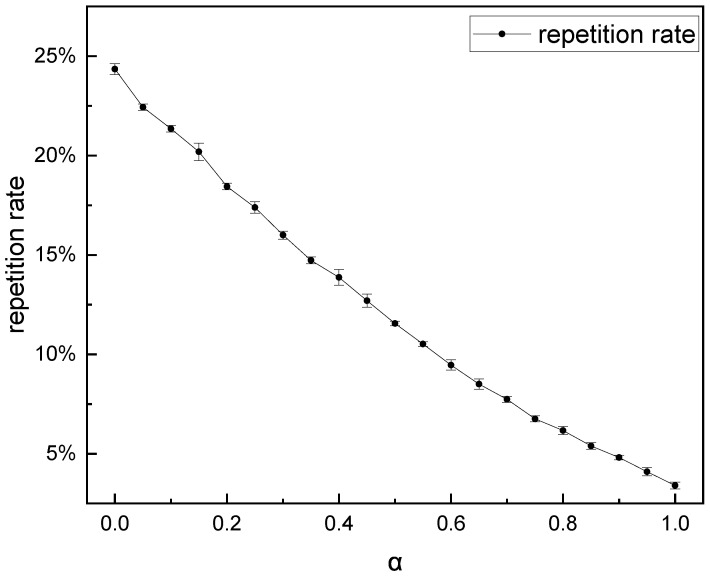
The repetition rate of $10^{7}$ passwords with different hyperparameter α.

**Figure 6:**
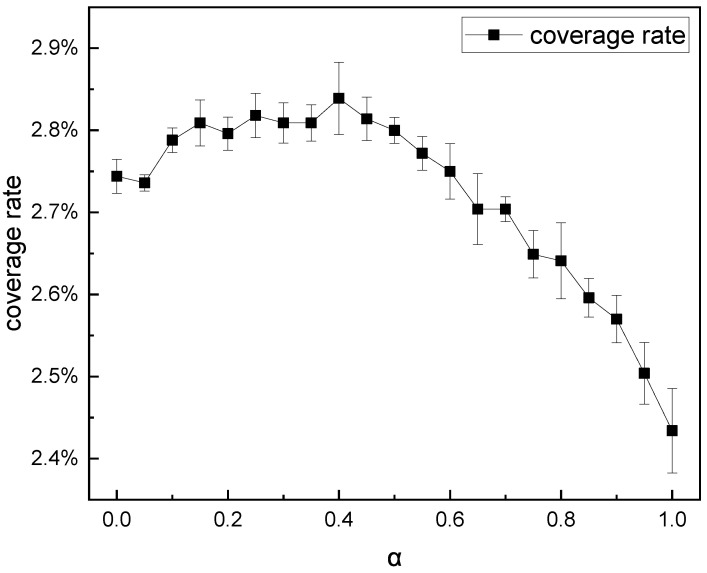
The coverage rate of $10^{7}$ passwords with different hyperparameter α.

To analyze the influence of the password-probability label-learning method on password generation, we must first experiment with the hyperparameter α value and confirm the optimal value. We used the five-fold cross-validation method to determine the optimal value of α. As mentioned in Section 3.2, these α take on value at an interval of 0.05, [0, 1]. By comparing the repetition rate and the coverage rate of the passwords generated by these models with different α, we could find the best performance model and the α value at that point. All models had the same parameters except α and trained the same steps under the same learning rate. After we generated $10^{7}$ passwords in each model, the experimental results are shown in Figure 5 and Figure 6.

As mentioned in Section 3.2, we aimed to generate passwords that were as unique as possible for more efficient password cracking and password security evaluation. However, these did not conform to the characteristics of the training set. From the result in Figure 5, we can see that the repetition rate drops sharply compared to the repetition rate of PassTCN in Table 4, which means that the password probability label learning method can substantially reduce the repetition rate of the generated password. We also can see that the repetition rate of the generated passwords decreases with the increase in α; this is because the effect of data repetition, represented by Equation (5), reduces with the increase in α. When α is equal to 1, all the passwords in the training set appear only once, meaning that each password has the same probability of occurrence. When α decreases, the probability of different passwords represented by the label through α differs, and high-probability passwords are more likely to be generated. This showed that the network trained with password probability label learning can learn the probability distribution of passwords in the training set.

From the result in Figure 6, we can see that the functional relationship of the coverage rate and α is not monotonous. When α=0.4, the average hit number of passwords is 56,844, and the coverage rate reaches the maximum value of 2.84%. Therefore, the optimal α is found and the value is set to 0.4 when we deal with the RockYou dataset. When α=1, the model is the same as the model using One-Hot Encoding, the training set deduplicates, and the coverage rate is 2.43%, as shown in Figure 6. This indicates that using the password’s probability label learning method to train the network can significantly reduce the repetition rate of the generated passwords and better learn the internal distribution of the password compared with the simple and crude deduplication of the training set (α=1).

Our work showed that the password probability label learning method couples the password’s probability distribution with the One-Hot label, and can effectively learn the intrinsic correlation of passwords.

### 4.4. Compare with Other Models

Based on the above experiments, the final model parameters in this paper are as follows: the total sequence length is 40, the valid sequence length is 20, and the hyperparameter α is 0.4. The above parameters were used to train the model in the 8:2 split of RockYou dataset, generating $10^{8}$ passwords. A comparison with other neural network models is shown in Table 5.

As shown in Table 5, PassTCN-PPLL achieved the best performance and has the highest coverage rate among all models in Table 5. When we generated $10^{8}$ passwords, the coverage rate of PassTCN-PPLL was 87.2% higher, and the repetition rate was 45.1% lower than that of PassGAN. The coverage rate increased by 42.9%, and the repetition rate diminished by 17.4% compared with FLA. Compared to the VAEPass, PassTCN-PPLL get a 72.6% higher coverage rate and a 31.7% lower repetition rate. Compared to OMEN, a classic and traditional password-guessing method that is used in practice, PassTCN-PPLL also obtains a 27.4% higher coverage rate. These experimental results prove the superiority of our model.

Comparing PassTCN-PPLL with PassTCN, we find that PassTCN-PPLL’s repetition rate has dropped sharply, but the promotion of the coverage rate is small. This means that the password-probability label-learning method can reduce the repetition rate without losing the probability distribution information of the training set. Moreover, reducing the repetition rate of the generated passwords slightly promotes the coverage rate.

Our proposed PassTCN can learn the training set features. Furthermore, our proposed password-probability label-learning method can significantly reduce the repetition rate of generated passwords without losing the training set information. Moreover, PassTCN-PPLL improved the coverage rate, although not by much. This also shows that we are in the right direction to reduce the repetition rate of password generation, especially when generating large-scale passwords.

## 5. Conclusions

This paper presents a password guessing model called PassTCN based on a temporal convolutional neural network. When generating $10^{8}$ passwords, the model has the best coverage rate compared to PassGAN and FLA. A generation model that uses sampling inevitably generates many duplicate passwords. In order to improve this shortcoming, we propose a label based on the password-probability distribution of the training set. This label can be combined with the One-Hot Encoding and the password’s probability distribution. This label learning method allows for us to deduplicate the training set without losing information. We used five-fold cross-validation method to determine the best value of the hyper-parameter α in the password’s probability label-learning method. Finally, we trained a PassTCN with the password-probability label-learning method on the RockYou dataset, called PassTCN-PPLL. The experiment showed that this method can effectively improve the coverage rate and reduce the repetition rate of password generation. As we know, password generation is a crucial technology for password-cracking and password security evaluation. In future work, we will focus on the practical use of this approach and instantiate the specific implementation as a password-checker or a password-cracker. Meanwhile, we will research a password-guessing model with a higher coverage rate on cross-site data in the following work.

## Figures and Tables

**Table 1:** The data of the 5-fold cross-validation.

Fold	Train		Validation	Validation-Train	
	Total	Unique	Total	Unique	Unique
1	19,953,819	9,651,744	4,988,454	2,909,052	2,003,456
2	19,953,819	9,653,713	4,988,454	2,906,118	2,001,487
3	19,953,819	9,654,946	4,988,454	2,904,866	2,000,254
4	19,953,819	9,652,840	4,988,454	2,908,011	2,002,360
5	19,953,816	9,652,658	4,988,457	2,907,580	2,002,542

**Table 2:** Experimental environment.

OS	Ubuntu 16.04.7
CPU	Intel(R) Xeon(R) Gold 5115 CPU @ 2.40 GHz
GPU	A TITAN XP graphics card
CUDA	CUDA 11.0
experiment framework	Pytorch 1.7.1

**Table 3:** The network architecture and number of parameters in each layer.

Layer		Inpout Shape	Output Shape	Number of Parameters
Embedding		(*, 40)	(*, 40, 256)	24,576
Residual block 1	conv1	(*, 40, 256)	(*, 40, 450)	345,600
	conv2	(*, 40, 450)	(*, 40, 450)	607,500
	conv3	(*, 40, 256)	(*, 40, 450)	115,200
Residual block 2	conv4	(*, 40, 450)	(*, 40, 450)	607,500
	conv5	(*, 40, 450)	(*, 40, 450)	607,500
Residual block 3	conv6	(*, 40, 450)	(*, 40, 256)	345,600
	conv7	(*, 40, 256)	(*, 40, 256)	196,608
	conv8	(*, 40, 450)	(*, 40, 256)	115,200
Dense		(*, 40, 256)	(*, 40, 96)	24,576

* represents the batch size in the training or generation process.

**Table 4:** The repetition rate and the coverage rate of PassTCN model compared with other models when $10^{8}$ passwords are sampled.

Model	Unique Number	Repetition Rate	Hit Number ^1^	Coverage Rate
PassTCN	46,240,992	53.8%	**293,830**	**12.28%**
OMEN [14]	**100,000,000**	**0.0%**	234,594	9.89%
FLA [18]	68,655,732	31.3%	211,077	8.82%
PassGAN [19]	52,815,412	47.2%	133,061	6.73%
VAEPass [20]	62,064,655	37.9%	160,589	7.30%

^1^ Hit number is the number of passwords in the testing set and not in the training set.

**Table 5:** The repetition rate and the coverage rate of each model when sampled $10^{8}$ passwords.

Model	Unique Number	Repetition Rate	Hit Number	Coverage Rate
PassTCN ^1^	46,240,992	53.8%	293,830	12.28%
PassTCN-PPLL ^2^	74,094,647	25.9%	**301,517**	**12.60%**
OMEN [14]	**100,000,000**	**0.0%**	234,594	9.89%
FLA [18]	68,655,732	31.3%	211,077	8.82%
PassGAN [19]	52,815,412	47.2%	133,061	6.73%
VAEPass [20]	62,064,655	37.9%	160,589	7.30%

^1^ PassTCN is the TCN model that trains on the origin training set and uses One-Hot Encoding. ^2^ PassTCN-PPLL is the TCN model that trains with the password-probability label-learning method.

## Data Availability

The dataset we used in this paper is the RockYou dataset, and it is openly available in https://ieee-dataport.org/documents/rockyou (accessed on 20 June 2022).
